# Clinical Management of Nonalcoholic Steatohepatitis (NASH) With the Use of Thiazolidinediones and the Additive Effect of Thiazolidinediones and a GLP-1 Agonist: Case Series

**DOI:** 10.7759/cureus.14082

**Published:** 2021-03-24

**Authors:** Valentina Rojas Ortiz, Jonathan Nieves, Enrique C Fernandez

**Affiliations:** 1 Internal Medicine, American University of Antigua, Osbourn, ATG; 2 Anesthesiology, Kendall Regional Medical Center, Miami, USA; 3 Family Medicine, Kendall Regional Medical Center, Miami, USA

**Keywords:** nash, pioglitazone, glp-1

## Abstract

We present five cases where patients were diagnosed with nonalcoholic steatohepatitis (NASH) and were treated pharmacologically. This is a common disease that is gaining clinical importance due to the long-term sequelae it may bring to a patient, such as cirrhosis, end-stage liver disease, and hepatocellular carcinoma. Diagnosis and treatment are crucial to make a difference in these patients. Diagnosis is mainly through obtaining alanine transaminase (ALT) and aspartate aminotransferase (AST) levels and excluding excessive alcohol use and other identified liver diseases. Diet and lifestyle are the first options in the treatment of NASH, but some pharmacotherapy has been tested for the cure of NASH. Insulin-sensitizing medications, such as Pioglitazone, have shown beneficial effects but with limited success and increase weight as a side effect. The GLP-1 receptor agonist, which are used in diabetes mellitus type two, has shown significant results in patients with NASH such as decreasing ALT levels, body weight, and hepatic fat.

## Introduction

Nonalcoholic fatty liver disease (NAFLD) is a common cause of chronic liver disease in the United States [1]. Although it can entail a complex interaction between genetics and environmental issues, the risk for NAFLD is increasing because more people are presenting with diabetes, obesity, and metabolic syndrome [2-3]. Nonalcoholic fatty liver disease (NAFLD) is described as a range of liver diseases, including nonalcoholic fatty liver and nonalcoholic steatohepatitis (NASH), and both have the possibility to develop a progressive liver disease [1]. Clinical importance is rising because this disease is associated with long-term sequels, such as cirrhosis, end-stage liver disease, and hepatocellular carcinoma [1]. Prompt diagnosis, timely referrals, and finding an effective treatment are crucial in making a difference in these patients [4]. Nonalcoholic fatty liver disease is usually asymptomatic or has nonspecific symptoms, for example, fatigue, right upper quadrant discomfort, or epigastric fullness. Commonly, nonalcoholic fatty liver disease is recognized in primary care physicians’ visits through abnormal liver chemistries or incidental ultrasound findings [1]. In theory, a liver biopsy should be contemplated, but it is rarely done [1]. Patients with elevated transaminases, excluding the history of excessive alcohol use and other identified liver diseases, should have NASH considered as one of the diagnoses [1]. There are no established screening regimens even in high-risk patients with metabolic syndrome or diabetes, therefore, obtaining random alanine transaminase (ALT) and aspartate aminotransferase (AST) levels is recommended.

NASH’s pathogenesis is not completely understood, but it has been demonstrated to have an association with insulin resistance and sensitization of the liver to metabolic injury [1-2,4]. NASH/NAFLD has been managed mainly throughout diet and lifestyle changes individualized to each patient, but it has been very unsuccessful due to the extent of weight loss needed to resolve liver changes [1]. There is a growing need for pharmacotherapy, but unfortunately, there is no Food and Drug Administration (FDA)-approved medication for the successful treatment of NAFLD/NASH, which is reversible [4-5]. Several insulin-sensitizing medications have been tested for the treatment of NASH, but they have shown limited success [4]. Pioglitazone has shown a good effect, reducing steatosis, inflammation, and fibrosis [1]. The natural antioxidant, Vitamin E, has been shown to alleviate oxidative stress suppressing lipid peroxidation [1]. In addition, a GLP-1 receptor agonist, such as exenatide and liraglutide, is used in diabetes mellitus type two, and several studies have shown significant results in patients with NASH such as decreasing serum ALT, improvement in hepatic fat, and fibrosis [6].

In this case series, there are five cases of patients with metabolic syndrome and a clinical diagnosis of NASH after excluding any other liver disease and using liver function tests. The effects of insulin-sensitizing medication in the treatment of NAFLD/NASH were observed for months. Medical records were used to review medical history, pharmacological management, and lab results. AST/ALT levels were compared prior to and after treatment was started. There was a decrease in AST/ ALT on follow-up labs in patients taking Pioglitazone. There was a possible advantage of combining insulin-sensitizing medications specifically Pioglitazone and a GLP-1 receptor agonist.

## Case presentation

Case 1 

A 55-year-old Hispanic male with a past medical history of hypertension and diabetes complaining of abdominal distension was seen in the clinic to review labs. At this time, he was taking losartan, HCTZ 12.5 mg QID, glipizide 10 mg BID, metformin 1000 mg BID, and Vitamin D 50000 units weekly. Regarding social history, he denied smoking, alcohol/drugs, and regular exercise. The physical exam was within normal limits, body mass index (BMI) was 33.76. and vital signs were stable.

After reviewing lab work, liver enzymes were elevated: AST (187) and ALT (94). At that time, there were no signs or symptoms of acute liver injury. After additional testing and imaging results, an additional diagnosis of NASH was made. The patient was placed on Pioglitazone 30 mg QID.

**Figure 1:**
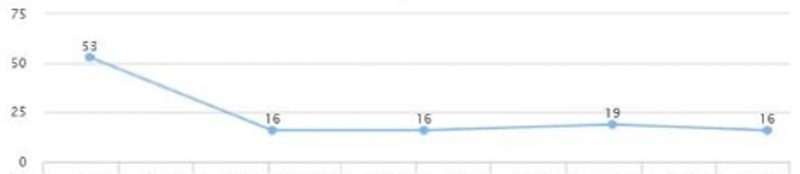
Case 1: AST AST: aspartate aminotransferase

**Figure 2:**
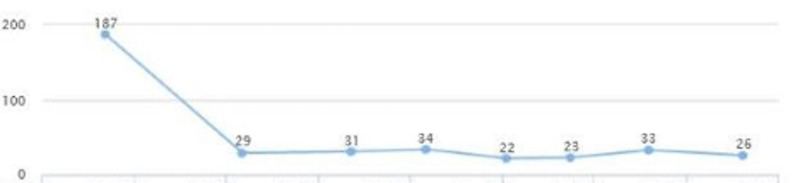
Case 1: ALT ALT: alanine transaminase

**Figure 3:**
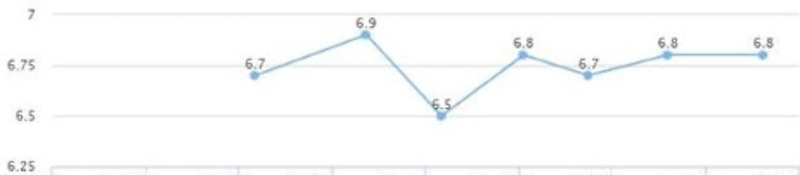
Case 1: HbA1c HbA1c: glycated hemoglobin

**Figure 4:**
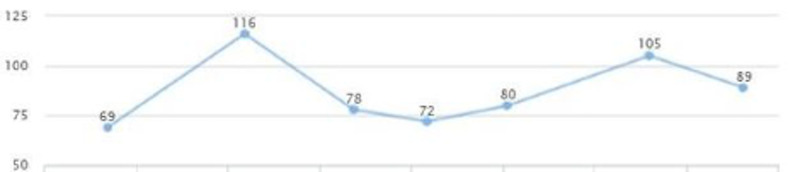
Case 1: Triglyceride

**Figure 5:**
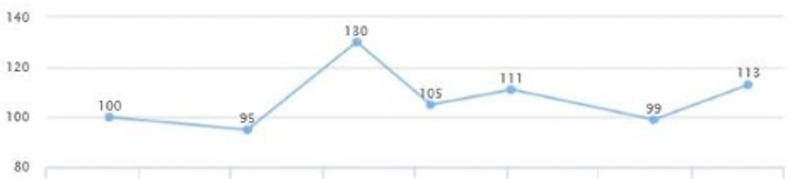
Case 1: LDL LDL: low-density lipoprotein

**Figure 6:**
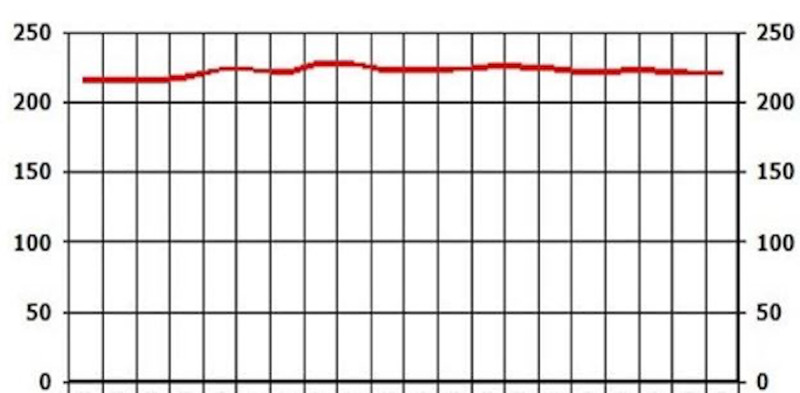
Case 1: Weight

On the follow-up visit (eight months later), liver enzymes were back within the normal range: AST of 25 and ALT of 29 (Figures 1, 2). These levels were sustained for multiple years without another episode. In addition, the patient's glycated hemoglobin (HbA1c) remained under seven, with a triglycerides level within the range of 69-116 and low-density lipoprotein (LDL) cholesterol of 95-130 over a span of three years (Figures 3, 4, 5). Although the patient reported lifestyle changes that included diet and increasing exercise, there was a slight increase in weight, BMI 34.56 (Figure 6).

Case 2

A 55-year-old female was seen in the clinic for an annual checkup. She had a past medical history of hypothyroidism and was currently on no medications. Regarding social history, she denied smoking, alcohol/drugs, and exercise. She was currently married and reported caffeine use. The physical exam was within normal limits, BMI was 34.57, and vital signs were stable.

**Figure 7:**
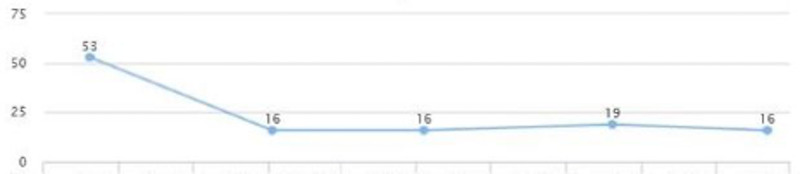
Case 2: AST AST: aspartate aminotransferase

**Figure 8:**
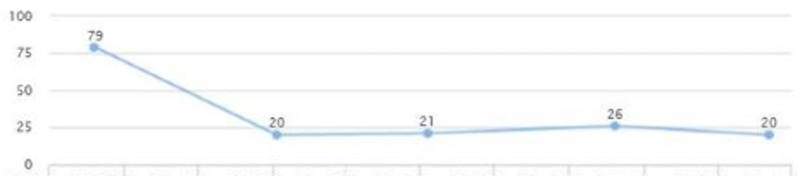
Case 2: ALT ALT: alanine transaminase

After reviewing lab work, liver enzymes were elevated: AST (187) and ALT (94) (Figures 7, 8). At this time there were no signs or symptoms present of acute liver injury. After additional testing and imaging results, the additional diagnosis of NASH and diabetes were made. The patient was placed on ramipril 2.5 mg QID and Pioglitazone 30 mg QID. 

**Figure 9:**
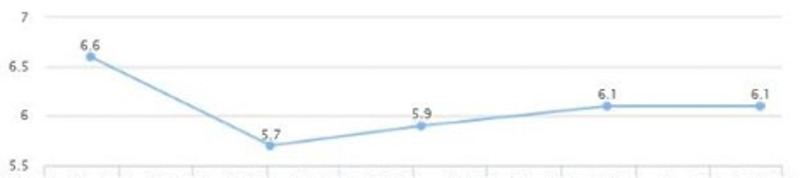
Case 2: HbA1c HbA1c: glycated hemoglobin

**Figure 10:**
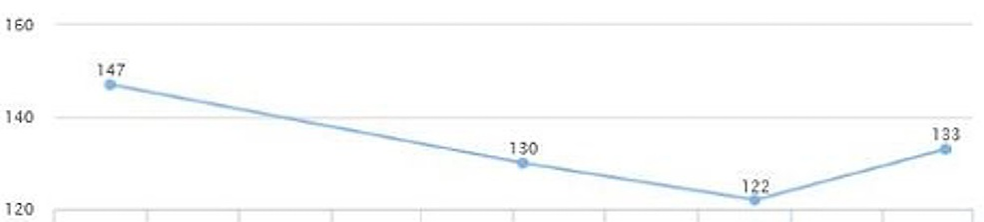
Case 2: LDL LDL: low-density lipoprotein

**Figure 11:**
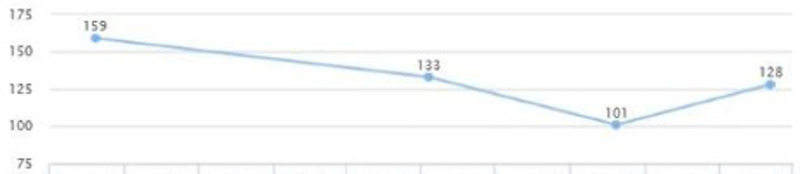
Case 2: Triglyceride

**Figure 12:**
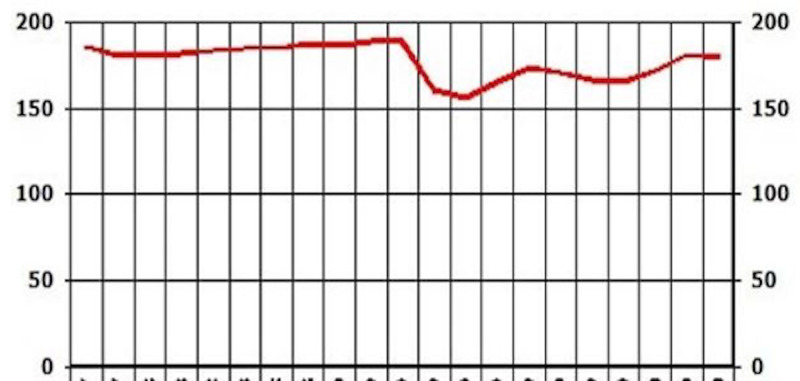
Case 2: Weight

On a follow-up visit (five months later), liver enzymes were: AST 16 and ALT 20; and the levels were sustained for over two years (Figures 7, 8). There was also a decrease in HbA1C from 6.6 to 5.7 in a five-month span (Figure 9). There was also noted a downward trend on LDL from 147 down to 122, triglycerides from 159 down to 101, and HDL cholesterol showed an upward trend from 47 to 61 in over a year's span (Figures 10, 11). There was a notable loss of weight that may be attributed to maintaining lifestyle changes and exercise (Figure 12).

Case 3

A 41-year-old male was seen in the clinic for follow-up, and he had a past medical history of hypertension, hyperlipidemia, and gastroesophageal reflux disease (GERD). The current medications were enalapril with HCTZ 10-25 mg one tablet QID and simvastatin 20 mg tablet in the evening. Regarding social history, he denied smoking and the use of illegal drugs. He also reported moderate alcohol use and caffeine. The patient was single. The physical exam was within normal limits, BMI was 32.93, and vital signs were stable with the exception of blood pressure (BP) of 156/101 mmHg.

**Figure 13:**
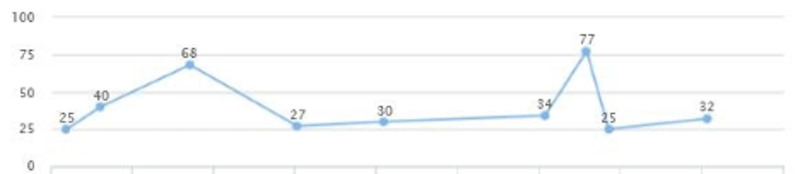
Case 3: AST AST: aspartate aminotransferase

**Figure 14:**
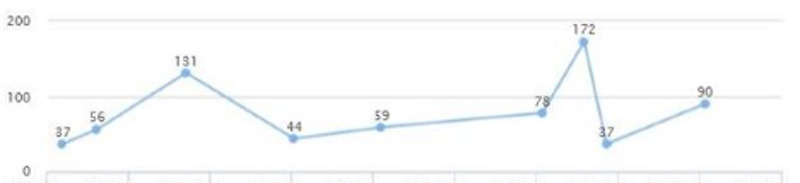
Case 3: ALT ALT: alanine transaminase

The patient was seen to follow up and review lab work. Liver enzymes were elevated: AST of 77 and ALT of 172 (Figures 13, 14). Fasting blood sugar was at the upper limit of normal and blood pressure was elevated. The additional diagnosis of prediabetes and elevated LFTs were made. The patient was placed on Pioglitazone 30 mg QID, and Cardizem 120 mg was added. 

**Figure 15:**
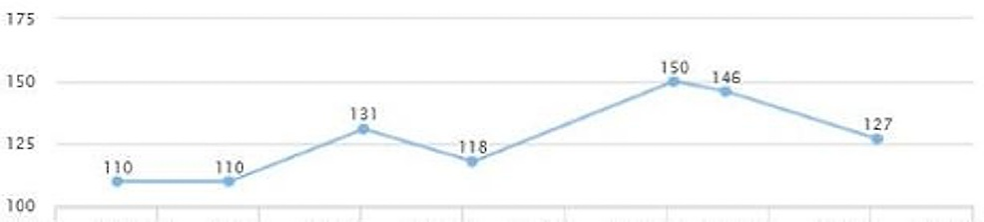
Case 3: LDL LDL: low-density lipoprotein

**Figure 16:**
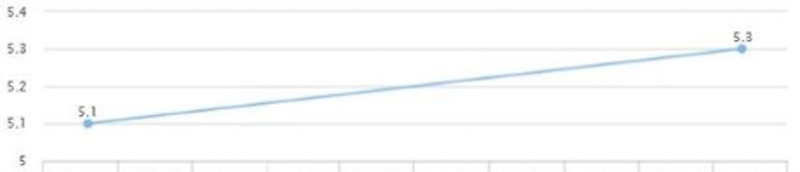
Case 3: HbA1c HbA1c: glycated hemoglobin

**Figure 17:**
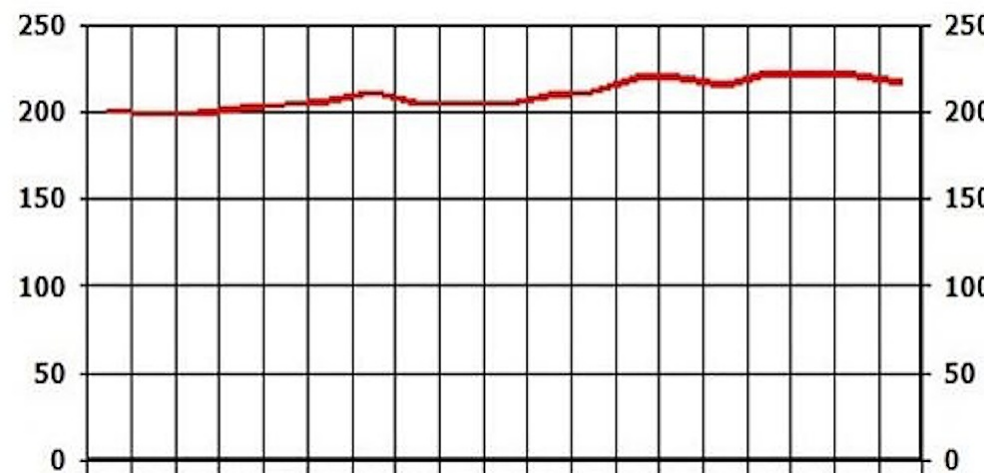
Case 3: Weight

On a follow-up visit after a couple of months, AST and ALT came down to 25 and 37, respectively (Figures 13, 14). A downward trend in LDL was also noted - from 150 down to 127 (Figure 15). HbA1c was maintained within the range of 5.1-5.3 (Figure 16). There was a slight upwards trend of weight (Figure 17). This could be attributed to the potential side effects of medication or noncompliance with medication and lifestyle changes.

Case 4

A 53-year-old female was seen in the clinic as a new patient with a prior diagnosis of type two diabetes, hypertension, and anxiety. Current medications were metformin 1000 mg BID, hydrochlorothiazide 12 mg BID, atenolol 50 mg BID, losartan 50 mg BID, and lexapro (escitalopram) 10 mg QID. Regarding social history, she denied smoking, alcohol/drugs, and exercise, reported caffeine use, and was currently divorced. Physical examination was within normal limits, BMI was 33.09, and vital signs were stable.

**Figure 18:**
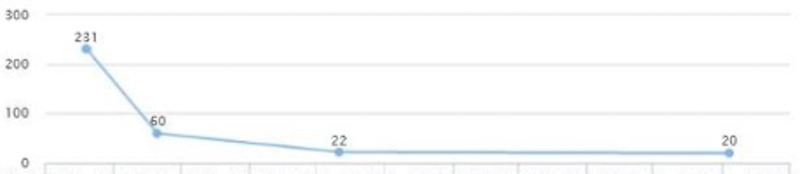
Case 4: AST AST: aspartate aminotransferase

**Figure 19:**
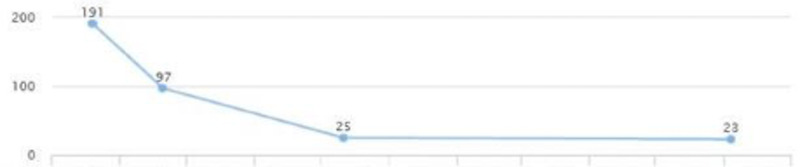
Case 4: ALT ALT: alanine transaminase

After reviewing lab work, liver enzymes were elevated: AST (231) and ALT (191) (Figures 18, 19). After additional testing and imaging results, an additional diagnosis of NASH was made. The new treatment plan included Vitamin E 400 units, Pioglitazone 15 mg QID, Invokamet (canagliflozin) XR 1000 mg two tablets, Ozempic (semaglutide) 2 mg subcutaneous weekly, hydrochlorothiazide 12 mg BID, atenolol 50 mg BID, losartan 50 mg BID, and Lexapro 10 mg QID, and metformin was stopped. 

**Figure 20:**
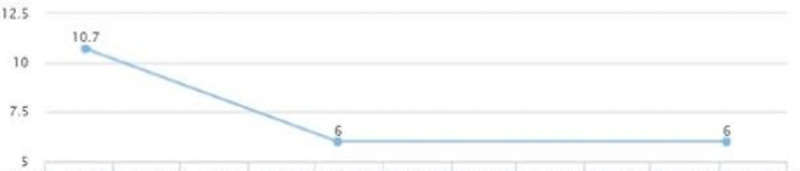
Case 4: HbA1c HbA1c: glycated hemoglobin

**Figure 21:**
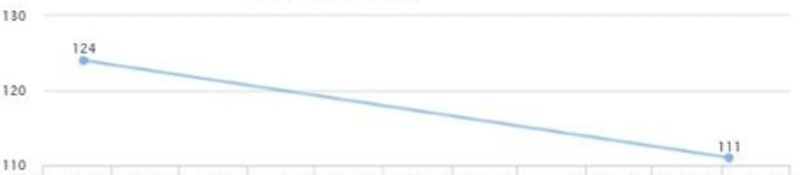
Case 4: LDL LDL: low-density lipoprotein

**Figure 22:**
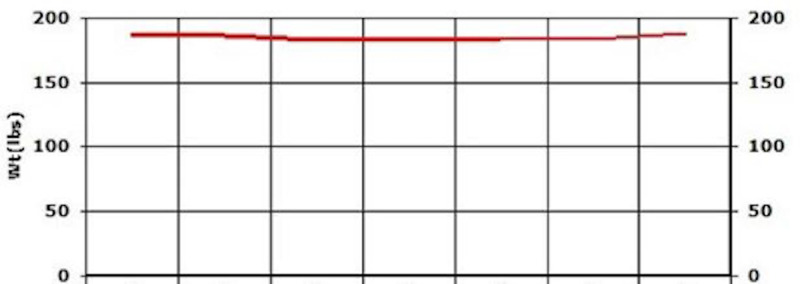
Case 4: Weight

The patient continued to follow up and showed a quick improvement in glycemic control (HbA1c came down from 10.7 to 6) (Figure 20). AST and ALT came down to 22 and 25 in three months (Figures 18, 19). LDL levels showed a downward trend as well, from 124 to 111 (Figure 21). The weight was maintained and the patient affirmed no lifestyle modifications (Figure 22). 

Case 5 

A 35-year-old male was seen in the clinic. The patient had a past medical history of essential hypertension, obesity, diabetes type two, vitamin D deficiency, hyperlipidemia, and NASH. Current medications were Vascepa 1 gm BID, Vitamin D 50000 weekly, atenolol-chlorthalidone 25 mg QID, Bydureon (exenatide) weekly subcutaneous injections, Pioglitazone 30 mg QID, and Vitamin E 400 units. Regarding social history, he denied smoking, alcohol/drugs, exercise, and use of caffeine. He was currently divorced. The physical examination was within normal limits, BMI was 39.38, and vital signs were stable.

**Figure 23:**
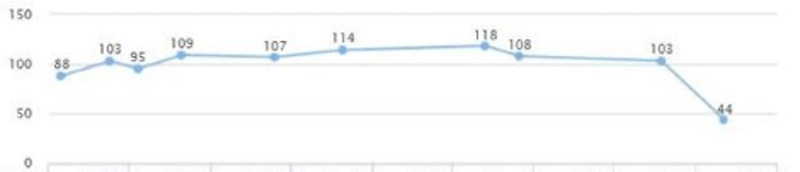
Case 5: ALT ALT: alanine transaminase

**Figure 24:**
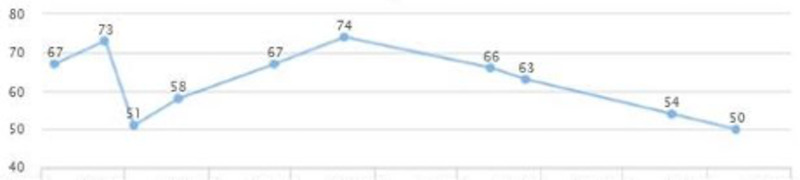
Case 5: AST AST: aspartate aminotransferase

The patient’s labs showed elevated liver enzymes and uncontrolled diabetes type two for over a span of three years following the treatment, although he reported being compliant with medication (Figures 23, 24). A plan was agreed to increase Pioglitazone to 45 mg QID.

**Figure 25:**
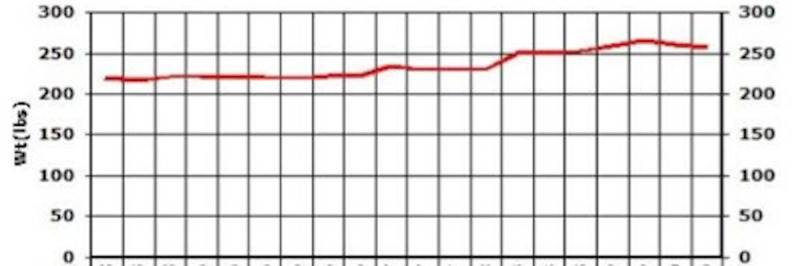
Cae 5: Weight

Repeated lab tests showed improvement in liver enzymes after a couple of months. AST and ALT came down to 50 and 44, respectively (Figures 23, 24). The patient reported that lifestyle modifications were not successfully maintained and weight was slightly increased (Figure 25).

## Discussion

In this case series, case numbers one, two, and three only had Pioglitazone as a treatment for NASH, and case numbers four and five had a combination therapy of Pioglitazone and a GLP-1 agonist. The last case needed an increase in the dose of Pioglitazone due to a resistant liver function test. All of them had a metabolic syndrome with obesity - BMI above 30. We observed that weight is a co-occurrence with NAFLD. Furthermore, understanding the hepatic effects of weight loss medications is critical [7]. In the Western world, an increase in weight and obesity has been the major repercussion for public health [4]. Complications of obesity such as cardiovascular and diabetic have been the primary focus, but possible hepatic effects have brought more medical attention to NASH [4].

Insulin-sensitizing medications, specifically, Pioglitazone, lowers the liver enzymes in patients with NASH [4]. Pioglitazone has some mechanisms of action that increase B-oxidation of the fatty acids and reduce the pro-inflammatory cytokines as well [1]. However, an increase in body weight is a significant associated side effect [4].

Furthermore, the hormone glucagon-like peptide one delays gastric emptying, suppresses appetite, and increases liver glucose uptake and peripheral insulin sensitivity [6]. GLP-1 agonists have shown some advantage in decreasing AST/ALT and are being associated with weight loss, making them a potential medication for use in patients with NASH and metabolic syndrome [6].

This study supports several pieces of research done concerning the use of thiazolidinediones for the treatment of NASH. The use of Pioglitazone showed positive results in all five patients, including a fast reduction in the liver function test and improvement in other laboratory markers such as HDL, LDL, triglycerides, and HgA1c, and in clinical symptoms such as fatigue and abdominal discomfort. Weight was maintained in most of the subjects and slightly increased in one (case number one), but they reported difficulty with lifestyle changes and incrementing exercise. Although diet and lifestyle changes are still the primary management of NASH, all patients reported difficulty accomplishing it. The need for pharmacotherapy is demonstrated and the use of combining insulin-sensitizing medications is an area that needs to be expanded on.

In the two patients with combined medication therapy (thiazolidinediones and GLP1 agonist), positive results were found, resulting in a faster reduction in liver function test in resistant patients and an improvement in other laboratory markers as well. Although a remarkable improvement in BMI and weight reduction was not found in these two patients, this could be due to non-compliance with medication or lifestyle changes. Positively, we did not find any side effects or patient discomfort from using both medications as compared with patients only using Pioglitazone.

## Conclusions

In conclusion, NAFLD/NASH may be reversible, but only after following the correct treatment regimen. Unfortunately, there are no FDA-approved medications for successful treatment, and there is a gap of information in the area of using a combined medical therapy. Especially for patients with a history of obesity, metabolic syndrome, and NASH, GLP-1 and Pioglitazone show an additive glucose-lowering effect. Therefore, a combination of the two agents may be a valuable therapeutic method for the treatment of type 2 diabetes, obesity, and NASH.
